# Spooneyism

**Published:** 1854-02

**Authors:** 


*Spooneyism.*—A new semi-monthly journal, to be devoted "to ancient and modern spiritualism," is announced to be published in New York city, edited by Judge Edmonds and Dr. Dexter. The title has not yet appeared. We would suggest "Spooneyism," and offer the following contribution for the ancient department:

*April 3d*, 1836. After dinner we stayed to see a curious half superstitious scene acted by the Malay women (natives of islands of the East Indian archipelago.) A large wooden spoon *dressed in garments*, and which had been carried to the grave of a dead man, they pretend becomes inspired at the full of the moon, and will dance and jump about. After the proper preparations, the spoon, held by *two women*, became convulsed, and danced in good time to the song of the surrounding children and women. It was a most foolish spectacle; but Mr. Liesk, (an English resident,) maintained that many of the Malays believed in its *spiritual movements*. The dance did not commence till the moon had risen."—See Journal, &c., Charles Darwin, M. A., F. R. S., vol. ii., p. 250.

N. B. As it is gravely determined upon to beset congress to appoint a *scientific commission* to examine and determine upon the phenomena of what is termed spiritualism, why not, Mr. Marcy, send our unoccupied Japan expedition for Malay women, to be in and of this commission? T.

